# Pyroptosis: Mechanisms and Links with Fibrosis

**DOI:** 10.3390/cells10123509

**Published:** 2021-12-12

**Authors:** Zihao Song, Quan Gong, Jiawei Guo

**Affiliations:** 1 Department of Immunology, School of Medicine, Yangtze University, Jingzhou 434023, China; 202071810@yangtzeu.edu.cn; 2 Department of Pharmacology, School of Medicine, Yangtze University, Jingzhou 434023, China

**Keywords:** pyroptosis, fibrosis, GSDM family, IL-1β, IL-18, TGFβ, inflammasome

## Abstract

Fibrosis is responsible for approximately 45% of deaths in the industrialized world and has been a major global healthcare burden. Excessive fibrosis is the primary cause of organ failure. However, there are currently no approved drugs available for the prevention or treatment of fibrosis-related diseases. It has become evident that fibrosis is characterized by inflammation. In a large number of studies of various organs in mice and humans, pyroptosis has been found to play a significant role in fibrosis. Pyroptosis is a form of programmed cell death mediated by the N-terminal fragment of cysteinyl aspartate-specific proteinase (caspase)-1-cleaved gasdermin D (GSDMD, producing GSDMD-N) that gives rise to inflammation via the release of some proinflammatory cytokines, including IL-1β, IL-18 and HMGB1. These cytokines can initiate the activation of fibroblasts. Inflammasomes, an important factor upstream of GSDMD, can activate caspase-1 to trigger the maturation of IL-1β and IL-18. Moreover, the inhibition of inflammasomes, proinflammatory cytokines and GSDMD can prevent the progression of fibrosis. This review summarizes the growing evidence indicating that pyroptosis triggers fibrosis, and highlights potential novel targets for antifibrotic therapies.

## 1. Introduction

Fibrosis is an intrinsic element in the pathogenesis of chronic and persistent injury [1]. However, excessive fibrosis can seriously affect the physiological function of the tissue. Fibrosis can develop in any one organ or in numerous organs. Fibrosis is responsible for approximately 45% of deaths in the industrialized world because no drugs are available for treating fibrosis, according to reports [2,3]. In both acute and chronic diseases, repeated damage to tissue often leads to the activation of fibroblasts that secrete extracellular matrix, eventually resulting in irreversible tissue damage. TGFβ is a key factor in the activation of fibroblasts [4], which are the main cells that secrete extracellular matrix. Fibroblasts differentiate into myofibroblasts, which participate in the formation and deposition of matrix when they are activated. Myofibroblasts secrete a broad spectrum of lipid mediators, chemokines, cytokines and reactive oxygen species (ROS) [5]. In addition, some other types of cells, such as hepatic stellate cells, mesangial cells, mesenchymal cells, epithelial cells and endothelial cells, also have the potential to become myofibroblasts. Notably, embryonic tissue studies have shown that tissue can heal without the development of a scar before the onset of the inflammatory response, suggesting that inflammation may be one of the causes of fibrosis [6].

Pyroptosis is associated with innate immunity [7], which plays a significant role in tumour immunity [8], infectious diseases [9], metabolic diseases [10] and nervous system-related diseases [11]. Pyroptosis is a form of programmed cell death (PCD), characterized by cell swelling and lysis, and resulting in the release of contents and the formation of inflammation that is mediated by gasdermin (GSDM) family proteins [12,13]. Inflammasomes are important upstream proteins of gasdermin D (GSDMD), a member of the GSDM family that is a pyroptosis executioner. Inflammasomes are composed of pattern-recognition receptors (PRRs) and cysteinyl aspartate-specific proteinase (caspase)-1, which is cleaved into the caspase recruitment domain (CARD), namely the P20 and P10 regions, after stimulation [12]. The P20/P10 tetramer, i.e., the activated caspase-1, can process GSDMD into the N-terminal fragment of GSDMD (GSDMD-N) and cleave pro-interleukin (IL)-1β and pro-IL-18 into mature IL-1β and IL-18, eventually leading to canonical pyroptosis and inflammation [14]. Moreover, there are numerous reports illustrating that pyroptosis is positively associated with fibrosis and inflammasomes [15,16]. This review intends to discuss the role and mechanism of inflammasomes and pyroptosis and further analyse the feasibility of using the GSDM family as a potential novel therapeutic target in the clinic.

## 2. Inflammasomes

Innate immunity is the first line of defence for detecting the presence of microbes and initiating reactions to eliminate potential threats. Invariant microbial motifs are detected through the germline-encoded PRRs engaged by the innate immune system. PRRs are usually expressed in dendritic cells, macrophages, monocytes, neutrophils and epithelial cells, serving as indicators of infection. Most PRRs can be classified into two main classes: membrane-bound receptors and unbound intracellular receptors. These receptors consist of five families based on their protein domain homology. Membrane-bound receptors include toll-like receptors (TLRs) and C-type lectin receptors (CLRs), which are located on the cell surface or in endocytic compartments. These receptors scan the extracellular milieu and endosomal compartments for pathogen-associated molecular patterns (PAMPs). Nucleotide-binding domain, leucine-rich repeat (LRR)-containing (or NOD-like) receptors (NLRs), RIG-I-like receptors (RLRs), and AIM2-like receptors (ALRs) are unbound intracellular receptors that are found in the cytoplasm, where they provide cytosolic surveillance. NLRs are distinct from those described above, recognizing not only PAMPs, but also host-derived danger signals (danger-associated molecular patterns, or DAMPs) [12]. NLRs with a CARD can associate with the CARD of pro-caspase-1, and lead to the establishment of complex results in the formation of inflammasomes, such as the NLRP1 inflammasome. NLRP1 was the first NLR identified to constitute an inflammasome complex. However, NLRs without a CARD can interact with pro-caspase-1 to form inflammasomes with the help of apoptosis-associated speck-like proteins containing a CARD (ASC) protein, as is the case for the NLR family pyrin domain-containing 3 (NLRP3) inflammasome. In the presence of inflammasome activation, activated NLRP recruits PYD-CARD-containing ASC proteins through a PYD–PYD interaction, and the CARD in ASCs subsequently combines with the domain of caspase-1. The NLRP3 inflammasome has been shown to be the most prominent NLRP inflammasome [17].

One of the most important functions of inflammasomes is sensing and recognizing PAMPs and DAMPs. In general, an extensive spectrum of PAMPs and DAMPs can activate inflammasomes. NLRP3, lipopolysaccharide (LPS, a PAMP) and HMGB1 (a DAMP) bind with TLR4 to activate the NF-κB pathway, subsequently upregulating the expression of NLRP3, IL-1β and IL-18. Moreover, BRCC3 [18], STING [19] and ABRO1 [20] can be activated by NLRP3 agonists, such as ATP, to promote the deubiquitination and sensitivity of NLRP3. In addition, the release of K^+^ efflux [21], ROS accumulation [22], oxidized mtDNA [23], cardiolipin [24] and cathepsin B [25] activates NLRP3 inflammasomes. Interestingly, NLRP3 senses the ROS produced in the mitochondria and is activated because the ROS promote the dissociation of thioredoxin from the thioredoxin-interacting protein (TXNIP). Subsequently, TXNIP binds with NLRP3 and induces the formation of the active NLRP3 inflammasome complex by stimulating NLRP3 recruitment of ASC and caspase-1 [26]. Furthermore, strong stimulation of the cell contributes to lysosomal rupture and the release of cathepsin B, leading to the upregulation of NLRP3 and IL-1β, but treatment with a cathepsin B inhibitor can have the opposite effect [12,27,28]. In summary, NLRP3 inflammasome activation in response to these conditions has been linked to the inflammation associated with the downstream release of IL-1β and IL-18 and their maturation via cleavage by caspase-1.

## 3. The Mechanism of Pyroptosis

Pyroptosis is a novel pathway of PCD that occurs in parenchymal cells and nonparenchymal cells [29,30,31]. It is a form of PCD distinct from apoptosis. Pyroptosis is a rapid process, involving cell membrane rupture, water influx, cellular swelling, osmotic lysis and the release of cell contents, including some proinflammatory cytokines, such as IL-1β, IL-18 and HMGB1, which leads to an inflammatory response. Pyroptosis is also accompanied by organelle deformation, the cleavage of DNA and nuclear condensation [12]. Due to the above characteristics, both apoptotic and pyroptotic cells show positivity on terminal deoxynucleotidyl transferase dUTP nick-end labelling (TUNEL) assay and propidium iodide (PI) staining [32]. Pyroptosis was first discovered in studies of bacterial infection [33], in which bacteria were found to cause macrophage death, and caspase-1 played a crucial role in this process [34]. Subsequently, it was gradually clarified that the type of macrophage death caused by bacteria is distinguishable from apoptosis. With in-depth study, the understanding of pyroptosis has gradually developed from the original definition of caspase-1-dependent PCD to gasdermin (GSDM) family protein-dependent PCD. GSDM family proteins mainly include GSDMA, GSDMB, GSDMC, GSDMD, GSDME and DFNB59. Except for DFNB59, other GSDM family proteins have similar N-terminal structures associated with cell membrane oligomerization that form the pyroptotic pore [35,36]. Recent studies have proven that GSDMB, GSDMC, GSDMD and GSDME are involved in pyroptosis, while the roles of other GSDM family proteins remain to be clarified.

Several hypotheses for pyroptosis have been formulated. They can be summarized as follows: (1) caspase-1-dependent GSDMD-mediated pyroptosis (also known as the canonical pathway) [37]; (2) caspase-4/5/11-dependent GSDMD-mediated pyroptosis (also known as the noncanonical pathway) [37,38]; (3) caspase-3-dependent GSDME-mediated pyroptosis [39]; (4) pyroptosis initiated by GSDMB and mediated by granzyme A (GZMA) [40]; and (5) pyroptosis initiated by GSDMC [41].

Canonical pyroptosis is mediated by inflammasome-activated caspase-1. Activated caspase-1 can be autocleaved into its CARD domain and P20/P10 dimers at the indicated sites. Afterwards, the two P20/P10 dimers oligomerize to form tetramers, executing their cleavage activity. The P20/P10 tetramers accurately bind to the GSDMD-C structural domain with high affinity by recognizing the specific site at which to cleave GSDMD in a mode similar to a key–lock mechanism [42]. Moreover, GSDMD is cleaved into the segmented N-terminal fragment, which can attach to the cell membrane and oligomerize to form the pyroptotic pore. Additionally, the P20/P10 tetramers can cleave pro-IL-1β and the pro-IL-18 transcribed as a result of NF-κB signalling into mature IL-1β and IL-18. In addition, the pyroptotic pore can cause the cell to swell because of the water influx induced by osmotic pressure, and IL-1β and IL-18 can escape through the GSDMD pore, leading to a proinflammatory response [37].

The noncanonical pathway of pyroptosis is mediated by caspase-4/5/11, of which caspase-4/5 are present in humans and caspase-11 is present in mice [38]. In the following, mouse caspase-11 will be used as an example. The caspase-11 stimulated by LPS cleaves GSDMD in the same manner as caspase-1 cleaves GSDMD [42,43]. However, caspase-11, unlike caspase-1, cannot cleave IL-1β and IL-18 [14]. Furthermore, caspase-11 can cause the cleavage of pannexin-1, causing the release of ATP, and the subsequent activation of the purinergic receptor P2X ligand-gated ion channel (P2X7) can also induce pyroptosis [44]. The nonselective pore channel also regulates the NLRP3 inflammasome activation mediated by K^+^ efflux.

In 2017, Wang et al. reported that another member of the GSDM family of proteins, GSDME, can be cleaved by caspase-3 and that the released N-terminal fragment directly attaches to the membrane to form pores to give rise to pyroptosis [39]. GSDME is expressed in a large number of cells but silenced in tumour cells. In the presence of GSDME, tumour cells switch from the state of apoptosis that was initiated by tumour necrosis factor α (TNF-α) and cycloheximide (CHX) to pyroptosis. In contrast, GSDME^−/−^ mice are protected from the various types of tissue damage and weight loss induced by CHX. These results provide a new strategy for the treatment of cancer. Moreover, caspase-3 is not only a key molecule in apoptosis but also a major element in pyroptosis. These findings imply that the solo action of cleaved caspase-3 in apoptosis that has been reported in previous studies may be incorrect, especially with regard to DAMP- or PAMP-induced damage.

Recently, Zhou et al. made a breakthrough in GSDMB research [40]. They reconstituted the expression of GSDM family proteins in HEK 293T cells, which express no endogenous GSDM, and cocultured the cells with NK-92MI cells. The coculture results showed that the granzyme A (GZMA) secreted by natural killer (NK) cells could cleave GSDMB. Similarly to that seen for GSDMD and GSDME, the oligomerization of the GSDMB-N fragment can also lead to the formation of the pyroptotic pore on the membrane. Researchers further found that both NK cells and cytotoxic T lymphocytes could kill some cancer cell lines, such as OE19, WS837 and SKCO1, via GZMA, and that the process was regulated by interferon γ (IFN-γ) and TNF-α. GSDMB is expressed in various healthy tissues, particularly the digestive tract, which indicates that the GZMA–GSDMB pyroptotic axis may play a significant role in the gut immune response.

**Figure 1:**
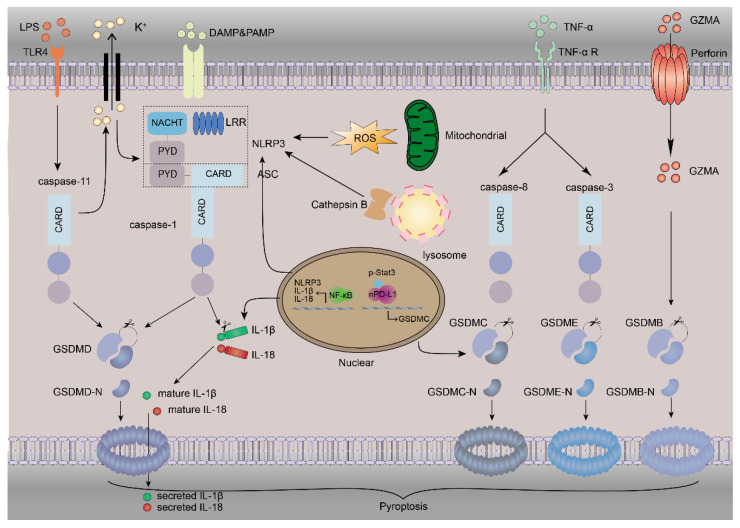
The mechanism of pyroptosis. Pyroptosis involves many proteins. NLRP3 inflammasome activation requires DAMPs or PAMPs to trigger the NF-κB-mediated upregulation of NLRP3, IL-1β and IL-18. The instigation of NLRP3 inflammasomes by K^+^ efflux increases the amount of ROS and the release of lysosomal cathepsin B, and subsequent caspase-1 activation leads to the direct cleavage of GSDMD, IL-1β and IL-18, inducing pyroptosis and inflammation. The binding of LPS to TLR4 stimulates the activation of caspase-11. Caspase-11 can also directly cleave GSDMD to release the N-terminal fragment of GSDMD (GSDMD-N), which can oligomerize to form pores and cause pyroptosis. This process can also cause NLRP3 activation by K^+^ efflux via nonselective pores. TNF-α binds with its receptor to activate caspase-3 and caspase-8. GSDME is cleaved by caspase-3, and GSDMC is transcriptionally upregulated by nPD-L1, which interacts with p-Stat3 in hypoxia and is cleaved by caspase-8 to induce pyroptosis. The GZMA released from cytotoxic lymphocytes and NK cells can enter the cytoplasm through perforin to cleave GSDMB, leading to pyroptosis.

Excitingly, Hou and colleagues found that nuclear PD-L1 cooperates with p-STAT3 and can bind to the promoter of GSDMC to promote GSDMC transcription under hypoxia [41]. Interestingly, the interaction of TNF-α with CHX stimulates caspase-8 to cleave GSDMC, resulting in the conversion of apoptotic cells into pyroptotic cells mediated by TNF-α and CHX, similar to that seen with GSDME (Figure 1).

Pyroptosis is a host defence mechanism that is of great immunological significance. A variety of GSDM family proteins have been found to induce pyroptosis and are widely used in the study of tumours, infections and other diseases. Further work needs to be done to establish whether pyroptosis could serve as a therapeutic target in the clinic.

## 4. The Role of Pyroptosis in Fibrosis

### 4.1. Liver Fibrosis

There are a variety of liver diseases that can stimulate liver fibrosis, such as non-alcoholic fatty liver disease (NAFLD), alcoholic liver disease (ALD), liver cancer and viral hepatitis. The final outcome of liver fibrosis is cirrhosis, resulting in portal hypertension and severe liver inflammation and greatly reducing the physiological function of the liver, which seriously affects people’s health and can even directly threaten their lives [1]. Hepatic stellate cells (HSCs) are crucial cells in the fibrogenesis of the liver. Once activated, HSCs can transform from quiescent, vitamin A-storing cells into myofibroblasts, which directly participate in the process of liver fibrosis [45]. As one of the markers of HSC activation, cysteine-cysteine chemokine ligand 5 (CCL5) initiates the activation of HSCs through the ERK activation of inflammasomes and the upregulation of IL-1β and TGFβ [46,47]. A large number of studies have shown that pyroptosis plays an important role in the process of liver fibrosis [29,48]. What is less clear is whether the nature of inflammasomes and proinflammatory cytokines greatly influences the process of fibrosis. Next, we will summarize the mechanisms that cause HSC activation and liver fibrosis from the perspective of inflammasomes and proinflammatory cytokines.

Studies have shown α-SMA and NLRP3 double positivity in human liver fibrosis specimens, where the expression level of NLRP3 is significantly increased compared with that in normal liver tissues, suggesting that the NLRP3 inflammasome is involved in the regulation of hepatic fibrosis [49]. In addition, the increased expression of inflammasomes, such as the NLRP3 and absent in melanoma 2 (AIM2) inflammasomes, has been reflected in human specimens and animal models of NAFLD [50], ALD [51], hepatocellular carcinoma [52], hepatitis C virus infection [46] and other diseases. Moreover, NLRP3^−/−^ [53] and NLRP3^KI^ [32,50,54] mouse models have been shown to have a reduced or worsened degree of liver fibrosis, respectively. MCC950, an inhibitor of NLRP3, reduces the degree of fibrosis in methionine/choline-deficient (MCD)-induced non-alcoholic steatohepatitis (NASH) in mice [55]. This finding suggests that targeting the NLRP3 inflammasome as a therapeutic strategy for hepatic fibrosis can achieve effective results in animal models, and, as such, such a strategy is expected to be successful in patients in the future.

NLRP3 inflammasomes are regulated by a variety of factors. NLRP3, ASC and caspase-1 are expressed in HSCs and macrophages in liver tissue due to the ROS accumulation caused by weakened autophagy [56] and reduced mitochondrial function [57]. Elongation of very long-chain fatty acid family member 6 (ELOVL6) can regulate oxidative stress through the JNK pathway, increase the expression of members of the NLRP3/caspase-1/IL-1β axis and aggravate fibrosis [58]. FUN14 domain-containing protein (FUNDC1), a receptor that mediates mitochondrial autophagy, inhibits the NLRP3 and AIM2 inflammasomes and fibrosis through the JAK/STAT pathway in hepatocarcinogenesis [52]. P66Shc is a redox enzyme that mediates mitochondrial ROS generation. Zhao and colleagues found that p66Shc controlled the NLRP3/ASC/caspase-1/IL-1β/IL-18 axis by regulating ROS and caused the activation of HSCs [57]. Angiotensin II (Ang II) is the main effector molecule of the renin-angiotensin system involved in the formation of fibrosis [59]. Ang II stimulates NADPH oxidase 4 (NOX4)-derived ROS and mitochondria-dependent ROS accumulation to aggravate hepatic fibrosis by activating HSCs through the IL-1β/NLRP3/Smad pathway and the TLR4/MyD88/NF-κB pathway [49]. Ang II-mediated miR-21 can invoke the ERK/NF-κB and SMAD7/SMAD2/NOX4 pathways to activate collagen deposition and NLRP3 activation in HSCs by targeting SPRY1 and SMAD7, which are downstream targets of TGFβ and Smad signalling [60]. Moreover, Ang (1–7), an inhibitor of Ang II, can improve bile duct ligation (BDL)-mediated hepatic fibrosis by inhibiting NOX4-dependent ROS and upregulating NRF2/ARE expression to decrease oxidative stress and NLRP3. In addition to the regulation of NLRP3 by ROS, microRNA, endoplasmic reticulum (ER) stress and the gut microbiota are also involved in the regulation of NLRP3. Jemenez Calvente et al. [61] showed that miR-233 could negatively regulate NLRP3 and reduce the degree of fibrosis in high-fat diet (HFD)-induced NASH. Lebeaupin et al. [62] showed that Bax inhibitor 1, a negative regulator of ER stress, inhibited fibrosis by decreasing NLRP3/caspase-1/IL-1β axis and TLR4/caspase-11 axis activity through the Akt/GSK and IRE1α-SXBP1 pathways. The outgrowth of Gram-positive and Gram-negative bacteria causes cirrhosis via the activation of NLRP3/AIM2 but not NLRP1/NLR family CARD-containing 4 (NLRC4) [63,64].

NF-κB can increase the expression of NLRP3 in addition to the transcription of IL-1β and IL-18. These proinflammatory cytokines not only promote liver inflammation, but also affect the activation of HSCs. The role of IL-1β in fibrosis has attracted increasing attention. IL-1β has been shown to directly stimulate the collagen secretion by fibroblasts in a dose-dependent manner [26]. Similar to IL-1β, IL-18 can induce TGFβ and collagen expression and stimulate fibroblast proliferation and HSC activation [65,66]. Therefore, the blocking of proinflammatory cytokines has been used to induce the remission of fibrosis. Anakinra, an IL-1β antagonist, has been shown to be effective in alleviating rheumatoid arthritis [67], Kawasaki disease [68], heart failure [69], COVID-19 [70], diabetes [71] and other diseases. Moreover, Petrasek et al. [51] have shown that anakinra can inhibit inflammasome activation and alleviate fibrosis in ALD.

Caspase-1 can cause the cleavage and maturation of IL-1β and IL-18. Gaul et al. [50] cultured human primary HSCs and an HSC cell line (LX2) with a caspase-1 inhibitor, which effectively reduced the expression of α-SMA and ACTA2, suggesting that the differentiation of HSCs into fibroblasts was inhibited.

**Figure 2:**
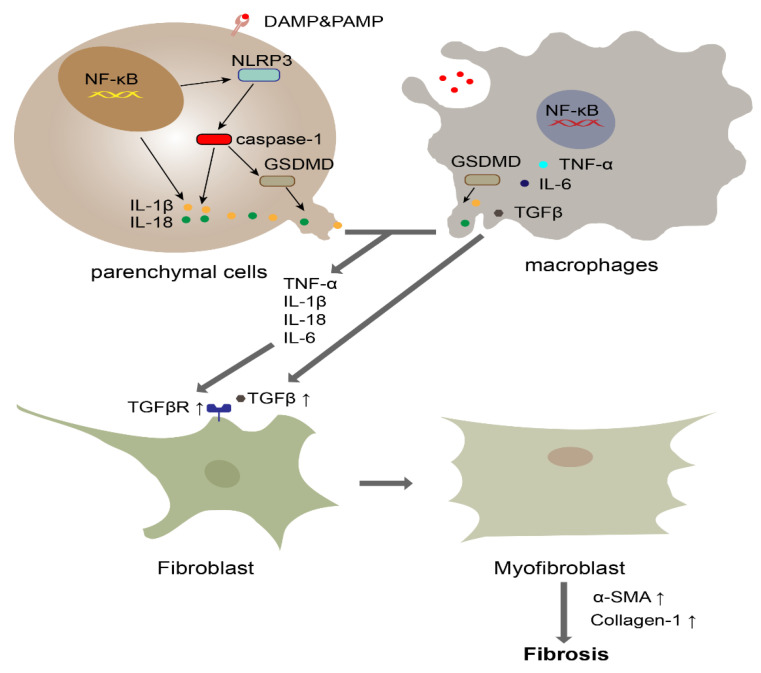
The role of pyroptosis in fibrosis. Macrophages and parenchymal cells, such as hepatocytes and alveolar epithelial cells, initiate the NF-κB pathway when they recognize DAMPs or PAMPs. Subsequently, upregulated NLRP3 activates caspase-1. Activated caspase-1 cleaves GSDMD into GSDMD-N, which oligomerizes to form the pyroptotic pore. The released IL-1β, IL-6, IL-18 and TNF-α promote the increased expression of the TGF receptor. Meanwhile, macrophages secrete TGF to cause the differentiation of fibroblasts into myofibroblasts. Subsequently, myofibroblasts induce substantial collagen deposition.

GSDMD is currently the most well-studied protein responsible for pyroptosis. GSDMD is cleaved by activated caspase-1 or caspase-11 into GSDMD-N, which subsequently causes pyroptosis and the release of cell contents, leading to an inflammatory response. The inflammatory response regulates the activation of HSCs and the secretion of collagen. Cenicriviroc, an inhibitor of CCR5, which is a chemokine receptor, can effectively alleviate liver injury and fibrosis by decreasing the cleavage of GSDMD, the release of IL-1β and the recruitment of TNF-α and IL-6 [72]. With regard to blocking GSDMD, Xu et al. [73] established a NASH model by feeding GSDMD^−/−^ mice an MCD diet for 8 weeks, and the results showed that liver steatosis, inflammation, and α-SMA levels were reduced compared with those of the control mice, who were fed an MCD diet. However, the overexpression of GSDMD-N induced by administering an adeno-associated virus (AAV)9-FLEX-GSDMD-N vector in Alb-Cre mice aggravated hepatic injury and enhanced NF-κB activity. These results suggest that GSDMD plays an important role in hepatic injury. In particular, GSDMD-N is more sensitive than GSDMD and is more likely to be a diagnostic marker for NASH (Figure 2).

In summary, inflammasomes and proinflammatory cytokines are involved in the process of fibrosis, and inhibiting them to improve fibrosis is a feasible strategy. More studies are needed to prove that inflammasomes can be used as a therapeutic target.

### 4.2. Kidney Fibrosis

Renal fibrosis is the most common ultimate pathological process resulting from chronic kidney disease (CKD). Fibrosis is also one of the most common causes of kidney damage. Similarly to their role in the liver, some proinflammatory and profibrogenic cytokines, including the IL-1β, IL-18, TNF-α and TGFβ that are secreted from resident and recruited immune cells, are also driving factors of renal fibrosis [74].

The role of NLRP3 in inflammasomes has been studied extensively; it is an important determinant in multiple kidney diseases, including unilateral ureteral occlusion (UUO) [75,76], crystal-induced renal injury [77], nephrocalcinosis-related chronic kidney disease [78] and metabolic syndrome-related kidney disease [79]. Overall, studies have illustrated the decreased expression of IL-1β, IL-18, α-SMA, collagen I and other fibrosis-related indicators in NLRP3^−/−^ mouse models of nephropathy, suggesting that NLRP3 participates in renal inflammation and fibrosis. The inhibitors of NLRP3 have been shown to work particularly well for kidney disease. MCC950 [74,77,80] and β-hydroxybutyrate [78] can effectively reduce the inflammatory response in renal tissue due to their critical effect on the reduction of TGFβ and α-SMA.

In addition to the NLRP3 inflammasome, many other inflammasomes have also been reported to be activated in renal inflammation and fibrosis. Luan et al. [81] found the activation of NLRC5 in the kidneys of STZ-induced diabetic mice and DB/DB mice, and studied the degree of renal inflammation and fibrosis through the NLRC5 knockout mouse model. NLRC5 was found to play an essential role in inflammation and fibrosis by regulating TNF-α/IL-6 and Smad2/Smad3/TGF-β through the NF-κB pathway. Komada et al. [76] found that both AIM2 and NLRP3 knockout effectively reduced renal collagen deposition and the fibrosis area 7 days after the establishment of a mouse UUO model. However, Schmidt-Lauber et al. [82] concluded that although treatment with an AIM2 ligand (DA:DT) increased IL-1β release, the release of IL-1β was dependent on NLRP3 rather than AIM2. In addition, there are many ways to affect inflammasome activation, and inflammasome regulation has also become a common research topic.

The IL-17 produced by CD4+ T cells can prompt fibrosis in different organs. IL-17 can stably increase the expression of TGFβR II on fibroblasts and enhance their sensitivity to TGFβ. On the one hand, caspase-1, NLRP3 and NF-κB have been demonstrated to be significant upstream activators of the IL17/TGFβ axis. On the other hand, the IL-17/IL-23 axis can adversely promote the activation of NLRP3. This finding suggests that the interaction between IL-17 and NLRP3 could constitute a positive feedback loop [83,84,85]. IL-36α is a novel member of the IL-1 family. Studies have shown that IL-36α is upregulated in both human and mouse renal tubular injury models and the renal tubular epithelial cells stimulated by HMGB1 or H_2_O_2_. IL-36α^−/−^ mice show a decreased expression of NLRP3/caspase-1/IL-1β/IL-18, IL-17/IL-23 axis members and NF-κB pathway members, which further reduces inflammation and fibrosis [83]. Oestrogens have been found to have a protective effect on mitochondrial function and to reverse the regulation of NLRP3/caspase-1/IL-18 and fibrosis. However, the application of oestrogen therapy has been limited by unfavourable adverse effects. Raloxifene, a selective oestrogen receptor modulator, has been demonstrated to be effective at reducing renal tubulointerstitial injury and fibrosis [86,87].

The canonical pathway of pyroptosis is mediated by caspase-1-dependent GSDMD-mediated pyroptosis. Activated caspase-1, by binding to inflammasomes, cleaves IL-1β and IL-18 to activate them and induce their release, thereby affecting the progression of fibrosis. The inhibitors of caspase-1 have also been used in animal models. Both Ac-YVAD-CMK [88] and VX-765 [80] have been shown to reverse inflammation and fibrosis of the kidney in vivo. Fluorofenidone can reduce the interaction between NLRP3 and ASC and the association of ASC with caspase-1, thereby reducing caspase-1 activation and IL-1β release and collagen deposition [89]. Recent studies have shown that TNF-α/caspase-3/GSDME-mediated pyroptosis in renal parenchymal cells is an important aspect of the tubule inflammation and fibrosis induced by ureteral obstruction, which is amplified by HMGB1. GSDME may also become a potential new target for the prevention and treatment of fibrosis [90].

### 4.3. Lung Fibrosis

The lungs are the core of the respiratory system and carry out the exchange of gases in the blood. The lungs are flexible to allow more or less air to be breathed in. However, a decrease in the elasticity of lung tissue resulting from the occurrence of fibrosis seriously affects the physiological function of the lung. Many studies indicate that inflammasomes and inflammatory cytokines are involved in the development of pulmonary fibrosis. In the lung injuries caused by silicon, asbestos, mechanical stretch, idiopathic pulmonary fibrosis (IPF) and cystic fibrosis (CF), there is a positive correlation between inflammasomes, inflammatory cytokines and fibrosis [27,91,92,93,94]. Both IL-1β and IL-18 promote the activation of fibroblasts [95]. ATP, ROS and cathepsin B, as upstream signals of inflammasome activation, may also serve as points to inhibit lung injury and fibrosis. Studies have shown that NADPH oxidase can participate in the destruction of lysosomes in macrophages, and that NADPH can transfer electrons through the plasma membrane to produce ROS [96,97]. ROS production and cathepsin B release, with both ROS and cathepsin B being agonists of NLRP3, promote the occurrence of pulmonary inflammation and the formation of fibrosis. Moreover, Ang II can contribute to the formation of fibrosis and the accumulation of NOX4-derived ROS and mitochondria-dependent ROS. Ying et al. [98,99] demonstrated that Ang II can stimulate the activation and collagen deposition of lung fibroblasts through the NLRP3/caspase-1/IL-1β pathway, and autophagy can clear ROS, abrogate the ubiquitination of NLRP3 and inhibit the expression of IL-1β to reverse Ang II-mediated pulmonary fibrosis. Anaerobic glycolysis [93] and aerobic glycolysis [100] are also thought to promote the formation of pulmonary fibrosis by activating NLRP3 or AIM2 to induce macrophage pyroptosis and by mediating the transcription of IL-1β through the HIF-α pathway, respectively. Anaerobic glycolysis mediates the innate immune response by activating NLRP3 and AIM2. Likewise, neutrophil aerobic glycolysis in CF stimulates the transcription of HIF-1α, with subsequent increases in IL-1β and NLRP3 transcription.

## 5. The Utility of Inflammasomes and Cytokines as Early Diagnostic Biomarkers of Fibrotic Disease and Therapeutic Targets

Fibrosis, as a chronic and persistent pathological injury of tissue, has become a global public health problem. Most studies show that fibrosis is a dynamic, multifactorial process [6]. Since most chronic diseases are important risk factors for tissue fibrosis and there are no drugs for the treatment of fibrosis, knowledge about early markers may facilitate the refinement of prevention strategies and provide useful tools for the diagnosis and treatment of fibrosis. In this regard, inflammasomes and proinflammatory cytokines have emerged as potential markers.

Indeed, clinical studies have demonstrated that inflammasomes, IL-1β, IL-18 and GSDM family proteins are all positively correlated with fibrosis in patients. Xu and colleagues [73] evaluated GSDMD and GSDMD-N levels in NASH and NAFLD patients and found that they were significantly different to the levels in healthy people. It was confirmed that GSDMD-N is more accurately measured in NASH patients than in NAFLD patients and may be a diagnostic marker for NASH. Similarly, caspase-1 was found to be positively associated with NASH, cirrhosis and IPF [50,63,101]. Canonical inflammasome activation, such as the activation of the NLRP3 and AIM2 inflammasomes, has also been reported to be involved in the progression of fibrosis [49,56,60,99]. IL-1β, IL-6, IL-18 and other proinflammatory cytokines in serum and tissues were noted to be significantly increased in fibrosis [63,83,100,102]. However, these studies include some limitations, such as small sample sizes and low numbers of samples of liver tissue, ascites and alveolar lavage fluid, which are not as readily available as blood samples. Therefore, larger sample sizes and more easily accessible samples, such as blood and urine samples, should be considered in future studies to further confirm the feasibility of inflammasomes, proinflammatory cytokines and GSDM family proteins as viable biomarkers for the early diagnosis of fibrosis.

TGFβ is a major profibrogenic cytokine and may serve as a potential antifibrosis target. However, the systemic inhibition of TGFβ has significant adverse effects [6]. Moreover, caspase-1, NLRP3 and NF-κB have been identified as important upstream activators of the TGFβ/SMAD axis [49]. Since pyroptosis is a critical part of fibrosis, antagonizing pyroptosis could reveal new therapeutic targets to counteract fibrosis. For example, the fibrosis area was significantly reduced in the NLRP3^−/−^ mouse model [103]. An inhibitor of NLRP3, MCC950, seems to show favourable antifibrotic effects [78,100,104]. The knockout of IL-1R, the administration of anakinra and the neutralization of IL-36R are characterized by reduced collagen deposition [51,83,104,105]. The use of caspase-1 inhibitors appears to attenuate inflammatory cells and α-SMA expression [74].

Most studies have found that the suppression of inflammasomes and IL-1β can restrain inflammation and fibrosis, but contradictory reports exist. For example, under the same stimulation conditions, NLRP3^−/−^ mice did not have a reduced fibrosis area and even had increased inflammation compared with control mice [53,106]. The use of anakinra did not improve fibrosis in a nephrocalcinosis-related chronic kidney disease mouse model [32,78]. It is worth noting that the majority of inflammasome inhibitors are used at the earlier stages of the disease and most knockout mouse models replicate the beginning of the disease, which is significant in the prevention of fibrosis. However, though the inhibition of inflammasomes prevents inflammation, the elevated levels of TGFβ and α-SMA could not be reverted [77]. This result may be due to pre-existing inflammasome activation in inflammatory cells, such as dendritic cells. Even if NLRP3 is inhibited, inflammatory cells continue to secrete some proinflammatory cytokines until their death by pyroptosis. In addition, the detection of GSDMD, inflammasomes and cytokines is not specific, and the presence of GSDMD is not limited to the occurrence and development of fibrosis; it can be a marker of fibrosis-related diseases and thus can be used to prevent these diseases. In conclusion, early diagnostic markers of fibrosis-related diseases urgently need to be found.

## 6. Conclusion and Perspective

Fibrosis is responsible for up to 45% of all deaths in the Western world and has been a major global healthcare burden. Pyroptosis is a form of PCD and is an innate immune response characterized by inflammation, which regulates the activation of myofibroblasts. The activation of myofibroblasts can result in the continuous secretion of extracellular matrix and eventually fibrosis. Therefore, inflammation is rapidly becoming a key instrument in the occurrence and development of fibrosis-related diseases. There is a body of evidence that suggests that GSDMD, inflammasomes and proinflammatory cytokines are viable biomarkers for the early diagnosis of obesity and that therapeutic targets for the prevention of the occurrence and development of fibrosis in fibrosis-related diseases are feasible. However, additional studies are necessary to warrant application in clinical settings.
